# Ellipsoidal analysis of coordination polyhedra

**DOI:** 10.1038/ncomms14235

**Published:** 2017-02-01

**Authors:** James Cumby, J. Paul Attfield

**Affiliations:** 1 Centre for Science at Extreme Conditions and School of Chemistry, University of Edinburgh, West Mains Road, Edinburgh EH9 3JZ, UK

## Abstract

The idea of the coordination polyhedron is essential to understanding chemical structure. Simple polyhedra in crystalline compounds are often deformed due to structural complexity or electronic instabilities so distortion analysis methods are useful. Here we demonstrate that analysis of the minimum bounding ellipsoid of a coordination polyhedron provides a general method for studying distortion, yielding parameters that are sensitive to various orders in metal oxide examples. Ellipsoidal analysis leads to discovery of a general switching of polyhedral distortions at symmetry-disallowed transitions in perovskites that may evidence underlying coordination bistability, and reveals a weak off-centre ‘*d*^5^ effect' for Fe^3+^ ions that could be exploited in multiferroics. Separating electronic distortions from intrinsic deformations within the low temperature superstructure of magnetite provides new insights into the charge and trimeron orders. Ellipsoidal analysis can be useful for exploring local structure in many materials such as coordination complexes and frameworks, organometallics and organic molecules.

## Abstract (web summary)

Simple polyhedra in crystalline compounds are often deformed, so methods for analysing their distortions are useful. Here, the authors demonstrate that analysis of the minimum bounding ellipsoid of a coordination polyhedron provides a general method for studying distortion.

Symmetry group analysis provides a rigorous approach for quantifying distortions of coordination polyhedra but large numbers of modes are often present, for example, an MX_6_ octahedron has 3 rotations and 15 distortion modes. Continuous symmetry and shape distortion approaches provide some simplifications by parameterizing distortions as a numerical deviation from an idealized polyhedron, and by describing distorted structures in terms of similarity to more ideal polyhedral shapes^1^,^2^,^3^,^4^,^5^. However, all of these methods require an appropriate reference polyhedron which may be difficult to define for irregular polyhedra such as 5- or 7- coordinate complexes. As a much more simple approach, single parameter approximations to describe polyhedra and their distortions are useful, but commonly used functions based on M–X distances *d* such as Δ=*σ*^2^(*d*)/<*d*^2^> (where variance *σ*^2^(*d*)=<*d*^2^>–<*d*>^2^ and <> denotes an average) are not sensitive to angular distortions, while angular variance functions ignore distance variations^6^,^7^,^8^.

A simple and general method for analysing distortions of polyhedra that takes account of shape irrespective of coordination number and geometry is to use an ellipsoidal approximation. Vertices of high symmetry polyhedra lie on the surface of a sphere. Deformations of the polyhedra transform the enclosing sphere into an ellipsoid and in this study we calculate the minimum bounding ellipsoid (MBE) of a given coordination polyhedron, which is the ellipsoid of smallest volume that encompasses all atoms, as described in ‘Methods' section. The MBE can be calculated for any polyhedron, irrespective of coordination number and geometry. All geometric information for the polyhedron is reduced to the properties of the MBE, plus the displacement vector of the central atom relative to the ellipsoid centre for complexes lacking inversion symmetry. The principal ellipsoid radii are ordered *R*_1_≥*R*_2_≥*R*_3_. Their mean <*R*> is related to the polyhedron size and their variance *σ*^2^(*R*) or s.d. *σ*(*R*)=✓*σ*^2^(*R*) quantifies polyhedral distortion. Ellipsoid shape is represented by the parameter *S*=*R*_3_/*R*_2_−*R*_2_/*R*_1_ which has range −1≤*S*≤1 where oblate (axially compressed) and prolate (axially stretched) ellipsoids respectively have *S*<0 and *S*>0, and a sphere has *S*=0. This method allows the magnitude of distortions of different symmetry to be compared directly, as shown in Fig. 1 for tetragonally and trigonally distorted octahedra. Our ellipsoidal approach has not been elaborated previously, although a best fit ellipsoid method was suggested for defining the centre of irregular polyhedra^9^,^10^, and an MBE method was recently used to characterize thermal expansion in perovskite-type ABX_3_ formates^11^.

Taking published crystal structures of metal oxides as examples, we demonstrate below that ellipsoidal analysis based on fitting the MBE provides new insights into phase transitions in perovskites, and complex distortions and electronic orders in iron oxides. Our approach has the advantages of providing sensitivity to both distance and angle distortions and of enabling straightforward comparison of coordination polyhedra of any size and symmetry with many fewer parameters than full symmetry mode analysis.

## Results

### Critical variations at perovskite tilt transitions

Perovskites are an important class of materials with diverse uses including ferroelectrics, for example, BaTiO_3_, magnetics and spintronics, for example, La_1−*x*_Sr_*x*_MnO_3_, and photovoltaics, for example, (CH_3_NH_3_)PbI_3_ (ref. 12). The ideal AMX_3_ perovskite structure is cubic with cuboctahedral AX_12_ and octahedral BX_6_ coordinations for which the MBEs are spheres. An important structural aspect is the tendency of perovskites to form superstructures of long-range ordered tilts or rotations of the MX_6_ octahedra, and 15 simple tilt systems have been classified^13^,^14^,^15^. Most analyses use structural quantities related to the octahedral tilt angle (*φ*) as the order parameter for transitions between these phases.

LaAlO_3_ has one of the simplest and most studied perovskite transitions, between low temperature rhombohedral (space group *R*3*c*) and high temperature cubic (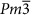
*m*) structures at *T*_c_=820 K. The *R*3*c* structure is formed by introduction of a single tilt with loss of some symmetry operations of the cubic phase and the transition is continuous (second order)^16^,^17^. Ellipsoidal analysis of the LaO_12_ and AlO_6_ polyhedra in LaAlO_3_ using published structural data^17^ shows that both ellipsoids change from spherical in the cubic phase to oblate in the *R*3*c* regime. Thermal variations of the ellipsoid radii and their average <*R*> and s.d. *σ*(*R*) are shown in Fig. 2.

Tilting of AlO_6_ octahedra deforms the LaO_12_ polyhedron and *σ*(*R*)_La_ is found to reproduce the temperature variation of the AlO_6_ tilt angle *φ*, which is proportional to *t*^½^ in the critical region approaching *T*_c_ (where *t* is the reduced temperature *t*=(*T*_c_−*T*)/*T*_c_) as shown in Fig. 2a. *σ(R)* values for the Al ellipsoid are an order of magnitude smaller but they provide a sensitive measure of AlO_6_ octahedral strain. This is predicted to vary with *φ*^2^ and hence show a ∼*t*^1^ critical variation^18^, as is observed in Fig. 2b. Hence ellipsoidal analysis provides a convenient and automatic way of accurately quantifying of the tilt and distortion of the AlO_6_ octahedra in LaAlO_3_, through *σ*(*R*)_La_ and *σ*(*R*)_Al_, respectively.

### Symmetry-disallowed transitions in perovskites

Although several branches of continuous symmetry descent from the cubic 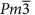
*m* structure are possible through successive tilting transitions, many investigated perovskites show symmetry-disallowed transitions. These have no group-subgroup relation between structures and hence are necessarily first order. Symmetry-disallowed transitions are relatively common in oxide perovskites, as shown in Supplementary Table 1, but the reason for their prevalence is unclear.

We have performed ellipsoidal analyses for many ABO_3_ perovskites where good quality structural data are available over multiple temperatures proximate to phase transitions. We use the ellipsoid shape parameter which has range −1≤*S*≤1, where negative and positive values respectively correspond to oblate and prolate distortions of *S*=0 spherical coordination. Symmetry-disallowed transitions between perovskite tilt superstructures are discovered to always change the sign of the shape parameter (*s*(*S*)=+ or −) for at least one of the two coordination ellipsoids (Fig. 3), whereas this is sometimes but not always observed for symmetry-allowed transitions. Prominent examples are below with further information in Supplementary Note 1 and data citations in Supplementary Table 1.

Orthorhombic-rhombohedral transitions are observed in many perovskites. RAlO_3_ perovskites (R=La, Ce, Pr and Nd) all display the 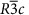
→
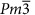
*m* transition described above for LaAlO_3_ (Fig. 3a,b and Supplementary Fig. 1). At lower temperatures, CeAlO_3_ and PrAlO_3_ also show a symmetry-disallowed orthorhombic *Imma*→rhombohedral 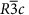 transition at which the shapes of both R and Al ellipsoids change, from prolate (*S*>0) in the *Imma* structure to oblate (*S*<0) in the *R*3*c* regime, as shown in Fig. 3a,b. Other ABO_3_ perovskites having this *Imma*→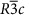 (BaCeO_3_) or a *Pnma*→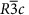 (PrNiO_3_, LaCrO_3_ and LaGaO_3_) symmetry-disallowed transition also display a sign change in at least one of the two ellipsoids as shown in Fig. 3c and Supplementary Table 1.

Symmetry-disallowed orthorhombic-tetragonal transitions are observed at high temperatures in a few perovskite materials. The *Imma*→I4/*mcm* transitions in BaPbO_3_ and BaTbO_3_ and the *Pnma*→I4/*mcm* transition in CaTiO_3_ all show a change of *s*(*S*)_B_ from positive to negative (Fig. 3d).

The famous perovskite BaTiO_3_ provides a striking example of *s*(*S*) changes. This material forms a series of ferroelectric phases where changes in the polarization defined by off-centre Ti displacement directions [111]→[110]→[100]→[000] (non-polar) gives rise to the *R*3*m*→*Amm*2→*P*4*mm*→
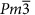
*m* sequence of phase transitions^19^,^20^. The first two transitions are symmetry-disallowed although in principle the magnitude of the polarization can vary continuously through them. Evolution of the off-centre Ti displacements and their local order through the transitions have been studied extensively^21^,^22^, but accompanying distortions of the TiO_6_ octahedron are less explored. Shape parameters *S*_Ba_ and *S*_Ti_ calculated from published structural data^23^,^24^ both show a striking switching behaviour through the *R*3*m*→*Amm*2→*P*4*mm* transitions in Fig. 3e,f. In contrast, the magnitudes of the off-centre displacements of the cations vary continuously through the transitions. Hence, the ellipsoidal shape parameters provide complementary information to the off-centre distortions. The former demonstrate abrupt changes in local coordination consistent with the first order transitions and further illustrate the general observation of sign switching at symmetry-disallowed changes, while the latter illustrate how the polarization magnitude effectively acts as a primary order parameter for the entire sequence of transitions.

The sign changes in ellipsoidal shape parameter at symmetry-disallowed perovskite phase transitions provide an important structural insight that has not been previously reported, even for much-studied materials like BaTiO_3_. The prevalence of sign changes suggests that there is a general bistability to the coordination polyhedra in tilted perovskite superstructures. In the simplest ellipsoidal approximation the distortions can be either prolate or oblate, and if these two states are of similar energy then changes of temperature or other tuning variables such as pressure or composition can lead to changes between them. Prolate and oblate distortions are generally accommodated by different tilt systems, resulting in frequent observation of symmetry-disallowed transitions in perovskites. The switching may be enhanced by electronic instabilities such as weak crystal field effects for Ce^3+^ and Pr^3+^ in RAlO_3_ or the second order Jahn–Teller instability of Ti^4+^ in BaTiO_3_.

### Iron oxide polyhedra

Ellipsoidal analysis provides a useful approximation for comparing the distortions and shapes of polyhedra with different coordination numbers and geometries through data-mining of crystallographic databases such as Inorganic Crystal Structure Database (ICSD) or Cambridge Structural Database (CSD) (refs 25, 26). These aspects are illustrated by using 499 iron oxide polyhedra from 390 structures in the ICSD where 98 Fe^2+^ and 401 Fe^3+^ sites have coordination numbers between 4 and 6. Results are shown in Fig. 4 and Supplementary Fig. 2, and further information is in Supplementary Note 2.

The plot of ellipsoid s.d. *σ*(*R*) versus mean radius <*R*> plot in Fig. 4a shows differing features for the common FeO_*n*_ coordinations. The distributions for FeO_4_ tetrahedra and FeO_6_ octahedra both have many points near *σ*(*R*)=0, reflecting their high ideal symmetries, and are clustered around <*R*>≈*d* where *d* is an average Fe–O distance for each coordination. Ideal values based on standard ionic radii for the predominant high spin Fe^3+^ state in tetrahedral and octahedral geometries are *d*=1.89 and 2.05 Å, respectively. Five-coordinate polyhedra are rarer and have less regular geometries, as evidenced by the bulk of the distribution lying between *σ*(*R*)≈0.1 and 0.3 Å. Highly anisotropic polyhedra have <*R*> values far from *d* and large values of *σ*(*R*). Ellipsoids describing FeO_4_ square planes have principal axes *R*_1_≈*R*_2_≈*d* and *R*_3_≈0 so <*R*>≈(2/3)*d* and *σ*(*R*)≈(✓2/3)*d*. The points at *σ*(*R*)>0.8 Å show this coordination, with a cluster of flat square planes at <*R*>≈1.3 Å and a more distorted variant at <*R*>=1.8 Å.

The plot of *σ*(*R*) versus ellipsoid shape *S* in Fig. 4b is another useful way to observe the spectrum of iron oxide polyhedra. *S* varies from near -1 for square planar complexes in the oblate distortion limit through many distorted and regular tetrahedra and octahedra at *S*≈−0.2 to 0.3. The V-shaped appearance of the distribution results from the most symmetric ellipsoid shapes imposing lower limits on σ(*R*); oblate coordinations (*S*<0) have *σ*(*R*)=−(✓2/3)*R*_2_*S* while prolate ellipsoids (*S*>0) have *σ*(*R*)=(✓2/3)*R*_2_*S*/(1-*S*). These limits are shown on the plot together with the line for geometrically scalene (triaxial) ellipsoids, which have *S*=0 but are non-spherical with *R*_3_/*R*_2_=*R*_2_/*R*_1_ but *R*_1_≠*R*_2_≠*R*_3._ This distortion is seen to be rather rare compared with oblate and prolate coordinations.

### Electronic orders in magnetite

Analysis of the coordination environment is important when subtle electronic features such as charge or orbital orders are present. A famous example is the iron oxide spinel magnetite, Fe_3_O_4_, where proposed Fe^2+^/Fe^3+^ charge order below the Verwey transition at 125 K has been controversial for many decades^27^,^28^,^29^,^30^,^31^,^32^,^33^,^34^,^35^. Conventional analysis of specific radial and tetragonal Jahn–Teller modes in the refined superstructure was recently used to demonstrate Fe^2+^/Fe^3+^ charge order and Fe^2+^ orbital order over 16 independent B-site FeO_6_ octahedra^33^. Short distances from Fe^2+^ sites to B-site neighbours further revealed weak direct Fe–Fe bonding effects that give rise to linear Fe_3_ units known as trimerons. Comparison of ellipsoidal parameter distributions for the 16 FeO_6_ octahedra in magnetite, which are constrained through connectivity and cooperative electronic distortions, against the large unconstrained set of six-coordinate iron oxide polyhedra from the above analysis of literature structures in Fig. 5 provides useful structural insights.

The s.d. *σ*(*R*) versus mean ellipsoid radius <*R*> plot for the literature FeO_6_ polyhedra in Fig. 5a has little structure other than a small displacement between the <*R*> histograms (which display the number *N* of polyhedra within each interval as log(*N*+1) to observe variations from *N*=0 to *N*>100) for Fe^2+^ and Fe^3+^ according to their ideal Fe–O distances based on ionic radii which are *d*=2.18 and 2.05 Å. However, the corresponding plot for magnetite (Fig. 5b) has a V-shape with the smallest Fe^3+^ and largest Fe^2+^ states having comparably large *σ*(*R*) values. This reflects the highly correlated nature of the distortions in magnetite, as each octahedron shares edges with six neighbours leading to an increase in *σ*(*R*) with increasing deviation in <*R*> from the overall structural average. However, instead of meeting with a symmetric V-shape, there is a significant excess in *σ*(*R*) for the Fe^2+^ line relative to that for the Fe^3+^ line where the two distributions meet at <*R*>=2.06 Å (corresponding to the average octahedral Fe–O bond length). This excess distortion provides direct evidence for the intrinsic Jahn–Teller distortion of octahedra containing triply-degenerate, high spin 3d^6^ Fe^2+^ whereas 3*d*^5^ Fe^3+^ is non-degenerate. The 0.03 Å magnitude of the excess in *σ*(*R*) is equivalent to a tetragonal compression where four coplanar Fe–O bonds expand by 0.02 Å and the other pair contract by 0.04 Å. Hence the ellipsoidal *σ*(*R*) versus <*R*> plot is valuable as it enables the single-ion electronic distortion due to Jahn–Teller instability for Fe^2+^ states to be separated from the correlated deformations of the octahedra due to their high connectivity in the magnetite structure. This is not easily done when using simpler measures of distortion such as the Δ parameter.

Magnitudes *D* of the Fe atom displacement relative to the ellipsoid centre are plotted against mean ellipsoid radius <*R*> in Fig. 5c. Neglecting a few outliers, the distribution has a peak at <*R*>≈2.0 Å (close to the average Fe–O distance for the predominant Fe^3+^ state) showing that many near-regular FeO_6_ polyhedra have off-centre instabilities. (Off-centre displacements might be expected to predominate in the most distorted octahedra, but the data do not support this expectation.) Octahedra at a crystallographic inversion centre have *D*=0 but there is a striking difference between the *D*>0 distributions for Fe^2+^ and Fe^3+^ states. Fe^2+^ displacements fall off with increasing *D* values up to a maximum value of 0.19 Å, whereas Fe^3+^ displacements range up to *D*=0.30 Å with a peak in the distribution at *D*≈0.15 Å. This disparity is greater than might be expected from crystal field effects (as *σ*(*R*) distributions for Fe^2+^ and Fe^3+^ in Fig. 5a are quite similar) and implies that another influence is significant. This is likely a weak ‘*d*^5^ effect' for the half-filled *d*-shell of Fe^3+^, similar to the much stronger *d*^0^ effect that gives rise to substantial off-centre displacements (second order Jahn–Teller distortions) for cations such as Ti^4+^ in BaTiO_3_ above. *d*^0^ distortions have previously been analysed by continuous shape measures^36^. Recent calculations have suggested that off-centre displacements are favourable for Fe^3+^ in trigonal bipyramidal coordination^37^, and they are of interest for possible multiferroics where both electric and magnetic polarizations are generated by the *d*^5^ configuration.

Linear variations of *D* with <*R*> are observed for both Fe^2+^ and Fe^3+^ states in low temperature magnetite in Fig. 5d, but with an excess displacement Δ*D*≈0.04 Å for Fe^3+^ ions where the two distributions meet at <*R*>=2.06 Å. Previous structural analysis showed that these Fe^3+^ displacements are associated with trimeron formation^33^,^35^, as Fe^3+^ ions move towards the spatially ordered lobes of *t*_2g_ orbitals of adjacent Fe^2+^ states resulting in weak Fe–Fe bonds. However, in light of the general analysis of Fe displacements revealed in Fig. 5c, the weak ‘*d*^5^ effect' may also enhance the Fe^3+^ displacements and hence assist trimeron formation in magnetite.

**Figure 1:**
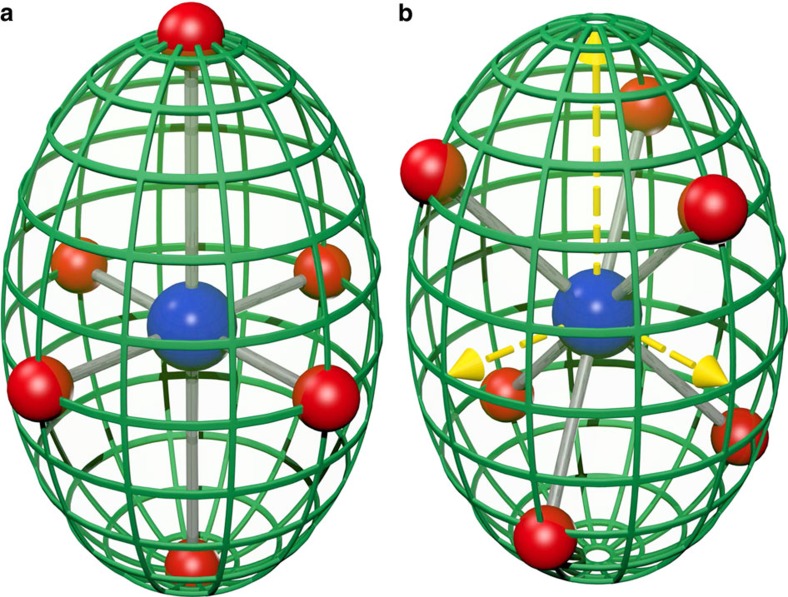
Ellipsoidal fits to distorted octahedra. (**a**) The fitted MBE (green net) for a tetragonally elongated MX_6_ octahedron (M/X=blue/red spheres) where M–X bond distances differ but *cis* X–M–X angles are 90°. (**b**) MBE fit to a trigonally distorted MX_6_ octahedron, where M–X distances are equal but *cis* X–M–X angles deviate from 90°. The two distorted octahedra have identical prolate MBEs with principal ellipsoid radii shown as yellow arrows in **b**.

The V-shaped distribution in the plot of s.d. *σ*(*R*) versus ellipsoid shape *S* for literature FeO_6_ polyhedra in Fig. 5e has lower limits on *σ*(*R*) corresponding to symmetric ellipsoid shapes as described above, and many polyhedra lie close to the oblate (*S*<0) or prolate (*S*>0) limits reflecting their high crystallographic symmetries. The corresponding plot for magnetite in Fig. 5f shows remarkable structure giving new insights into the electronic distortion. Points for the 16 FeO_6_ octahedra fall close to three lines parallel to the symmetric ellipsoid limits. The spinel lattice imposes a prolate trigonal distortion on the unique FeO_6_ octahedron in the high temperature cubic structure (like that shown in Fig. 1b), and this is broadened into a dispersion of distortions close to the prolate limiting line for the Fe^3+^ type states in the low temperature structure. The Fe^2+^ distribution is shifted parallel to the oblate limit, as tetragonal Jahn–Teller compression of Fe^2+^O_6_ octahedra gives oblate ellipsoidal distortion. However the points for these octahedra are highly structured and lie very close to two lines, one at fixed oblate distortion, showing dispersed trigonal distortions at constant Jahn–Teller deformation, and the other at a constant average trigonal distortion with varying Jahn–Teller effect. Further analysis of this distribution is required but it appears to be related to trimeron formation, as the three sites in each unit have similar prolate distortions as shown for the least and most distorted trimerons on Fig. 5f, and for all trimerons on Supplementary Fig. 3. This structural correlation within the octahedra forming each trimeron was not evident in previous analyses using conventional symmetry group analysis^33^. Overall, the *σ*(*R*) versus *S* plot provides an elegant separation of the single-ion electronic distortion, which is tetragonal and oblate, from the trigonal prolate deformations that are intrinsic to the distorted spinel lattice, with the two effects behaving as additive vectors parallel to the ellipsoid limits.

## Discussion

The above results demonstrate the utility of ellipsoidal analysis of coordination polyhedra. Our ellipsoidal approximation based on fitting the MBE has the advantages of providing sensitivity to both distance and angle distortions and of enabling straightforward comparison of coordination polyhedra of any size and symmetry with many fewer parameters than full symmetry mode analysis. Although the derived ellipsoidal parameters are not predicated on any particular type of distortion being present, they are shown to be highly sensitive to the orders of interest in the metal oxide examples presented.

Ellipsoidal analysis provides a convenient parameterization of octahedral tilts and strains in perovskites and has led to discovery of a general switching of polyhedral distortions at symmetry-disallowed transitions that may evidence underlying coordination bistability. This method is also useful for comparing large numbers of structures from crystallographic databases, here providing evidence for a weak off-centre ‘*d*^5^ effect' for Fe^3+^ ions that may be exploited in multiferroics. Ellipsoidal plots for the magnetite superstructure have enabled local electronic distortions to be separated from correlated lattice deformations, providing new representations of the complex electronic order for future analysis.

Coordination polyhedra are ubiquitous to chemical structure so ellipsoidal analysis is likely to prove an equally useful tool for probing coordination complexes and frameworks, organometallics and organic molecules, and could even be applied to larger scale packings of molecules or nanoparticles.

## Methods

### MBE fitting

The MBE for a given coordination polyhedron is calculated using a Khachiyan minimization algorithm^38^, resulting in three principal radii and a rotation matrix for the ellipsoid, and off-centre displacement *D* of the central atom. The principal ellipsoid radii are ordered *R*_1_≥*R*_2_≥*R*_3_. Their mean <*R*> is related to the polyhedron size and their variance *σ*^2^(*R*) or s.d. *σ*(*R*)=✓*σ*^2^(*R*) quantifies distortion. *S*=*R*_3_/*R*_2_ – *R*_2_/*R*_1_ describes ellipsoid shape. Values of <*R*>, *σ*(*R*) and *S* enable straightforward analysis and comparison of coordination polyhedra of any size and symmetry.

The MBE fitting algorithm is implemented within the PIEFACE (Polyhedra-Inscribing Ellipsoids For Analysing Crystallographic Environments) software package which uses Python 2.7. PIEFACE fits an ellipsoid to a set of points in Cartesian coordinates to within a supplied tolerance; if the points are coplanar then *R*_3_ is set to zero. The algorithm can be accessed directly or operated via simple graphical or command-line interfaces which allows direct input of CIF files, with optional control over parameters such as which atoms are coordinating, the maximum radius to use for coordination searching, and the fit tolerance. A number of other routines have also been implemented for data analysis within the PIEFACE package including output of ellipsoid parameters to a number of formats and interactive visualization of the resulting ellipsoids.

### Crystallographic data selection

Structural data were taken from published crystal structures. Where possible, structures obtained from neutron diffraction refinement were used to ensure accurate oxygen positions in the presence of heavy atoms. In the perovskite phase transition examples, variable temperature data were extracted from a single study wherever possible to avoid problems of systematic offsets between different experiments. For analysis of Fe oxide materials, polyhedra with Fe-coordinating atoms other than oxygen were rejected, as were mixed-cation sites with Fe occupations below 80%. Only ambient pressure and temperature structures were used.

### Code availability

Version 1.0.0 of PIEFACE used in this study is archived at doi:10.5281/zenodo.198370. Code updates are available at https://github.com/jcumby/PIEFACE.

### Data availability

**Figure 2:**
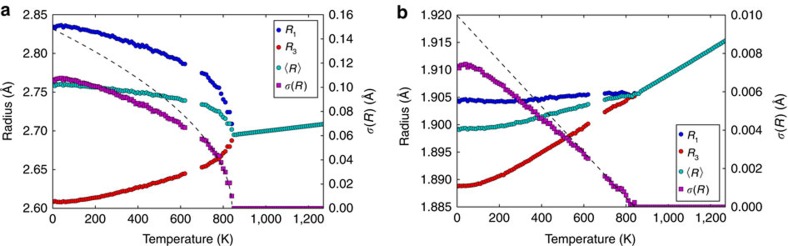
Ellipsoidal analysis of the 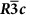
→
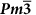
*m* structural transition in LaAlO_3_. (**a**) Variations of the ellipsoid radii, their average <*R*> and s.d. *σ*(*R*) for the LaO_12_ polyhedron, with a *t*^½^ fit to *σ*(*R*) points above 700 K shown. (**b**) Variations of the ellipsoid radii, <*R*> and *σ*(*R*) for the AlO_6_ octahedron showing linear *σ*(*R*) ∼*t* behaviour approaching the transition. *t* is the reduced temperature *t*=(*T*_c_−*T*)/*T*_c_.

**Figure 3:**
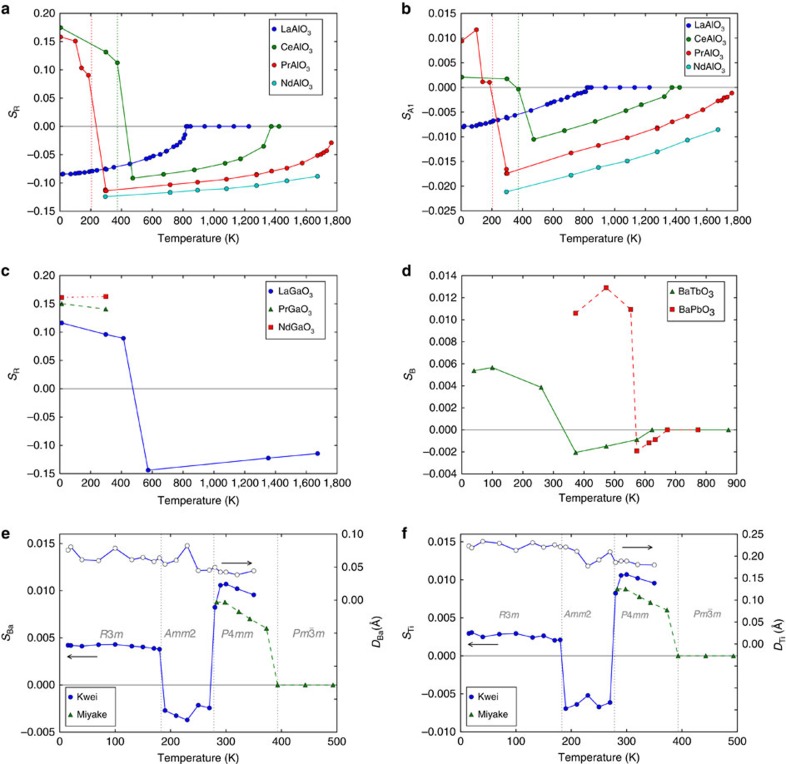
Thermal variations of the ellipsoidal shape parameter *S* for ABO_3_ perovskites. Changes of sign in *S* are observed at the symmetry-disallowed transitions. (**a**) *S*_R_ and (**b**) *S*_Al_ shape parameters for RAlO_3_ perovskites (R=La, Ce, Pr and Nd) with sign changes at the *Imma*→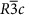 transition (denoted by vertical lines). (**c**) *S*_R_ variation in RGaO_3_ perovskites showing a sign change at the *Pnma*→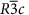 transition for LaGaO_3_. (**d**) *S*_B_ versus temperature for BaPbO_3_ and BaTbO_3_ through their *Imma*→I4/*mcm* transitions at 573 and 280 K respectively. (**e**) *S*_Ba_ and (**f**) *S*_Ti_ variations through the three structural phase transitions of BaTiO_3_ using published structural data from Kwei *et al*.^23^ and Miyake *et al*.^24^ Off-centre cation displacements *D* calculated from the former data are also shown.

**Figure 4:**
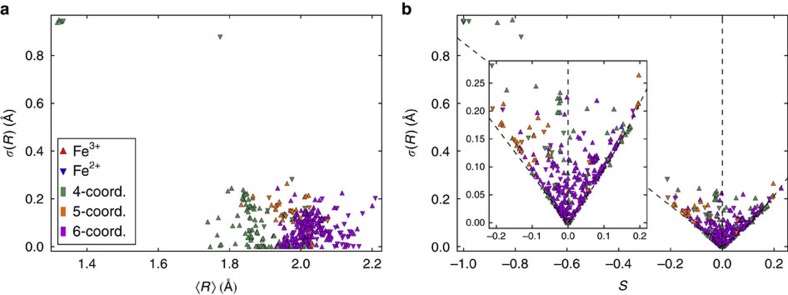
Ellipsoidal parameter plots for literature FeO_*n*_ polyhedra. 499 FeO_*n*_ polyhedra in iron oxides (98 for Fe^2+^ and 401 for Fe^3+^) taken from the ICSD with Fe coordination numbers *n* between 4 and 6 are shown. (**a**) s.d. *σ*(*R*) versus mean ellipsoid radius <*R*>. (**b**) *σ*(*R*) versus shape parameter *S* showing limits for oblate (*S*<0), geometrically scalene (*S*=0) and prolate (*S*>0) ellipsoids taking *R*_2_=1.81 Å. The inset (which does not obscure any points on the main plot) shows an expansion for the region near *S*=0.

**Figure 5:**
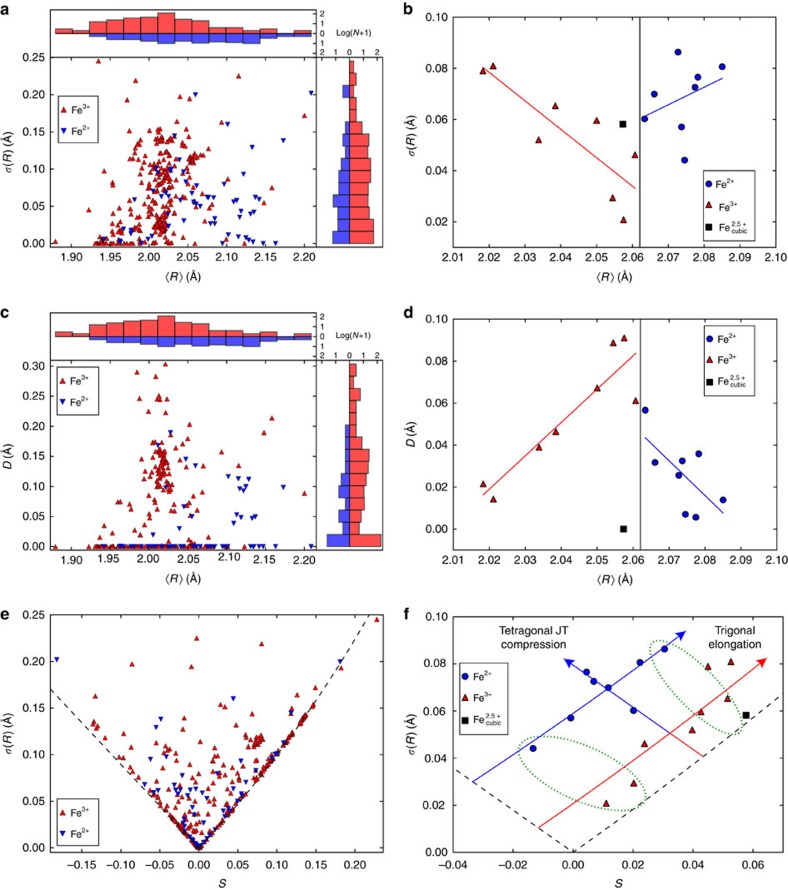
Ellipsoidal parameters for FeO_6_ polyhedra. The plots compare ellipsoidal parameters from iron oxide structures in the ICSD with those in magnetite. Histograms show distributions as log(*N*+1) for *N* polyhedra within each interval. Plots (**a**,**c**,**e**) describe 359 FeO_6_ polyhedra from 317 structures in ICSD. (**b**,**d**,**f**) are for the 16 independent B-site FeO_6_ octahedra in the reported magnetite superstructure at 90 K (ref. 33), and the single average Fe^2.5+^O_6_ octahedral site in the high temperature cubic structure at 130 K is also shown^28^. (**a**) s.d. *σ*(*R*) versus mean ellipsoid radius <*R*> for ICSD FeO_6_ structures. (**b**) *σ*(*R*) versus <*R*> for FeO_6_ octahedra in magnetite. (**c**) Displacements *D* of the cations from their ellipsoid centres versus <*R*> for ICSD FeO_6_ structures. (**d**) *D* versus <*R*> for FeO_6_ octahedra in magnetite. (**e**) *σ*(*R*) versus *σ*(*R*) versus shape parameter *S* showing limits for oblate (*S*<0) and prolate (*S*>0) ellipsoids for ICSD FeO_6_ structures taking *R*_2_=1.9 Å. (**f**) *σ*(*R*) versus *S* for FeO_6_ octahedra in magnetite, showing that points within the Fe^2+^ and Fe^3+^ distributions lie parallel to the prolate (trigonal elongation) and oblate (tetragonal Jahn–Teller compression) ellipsoid limits. Sets of three sites corresponding to the Fe^3+^–Fe^2+^–Fe^3+^ trimerons with the smallest and largest prolate distortions are encircled; assignments for all trimerons are shown in Supplementary Fig. 3.

All structural data used for ellipsoid fitting were taken from published crystal structures in the ICSD (version 2015.2)^25^, except for those for the variable temperature study of LaAlO_3_ shown in Fig. 2 which were taken fromref. 17.

## Additional information

**How to cite this article**: Cumby, J. & Attfield, J. P. Ellipsoidal analysis of coordination polyhedra. *Nat. Commun.* **8**, 14235 doi: 10.1038/ncomms14235 (2017).

**Publisher's note**: Springer Nature remains neutral with regard to jurisdictional claims in published maps and institutional affiliations.

## Supplementary Material

Supplementary Information Supplementary Figures, Supplementary Tables, Supplementary Notes and Supplementary References
