# Supplementary material for: Ellipsoidal analysis of coordination polyhedra
Source: Nat Commun. 2017 Feb 1;8:14235. doi: 10.1038/ncomms14235 (PMC5296646; doi:10.1038/ncomms14235)
Supplement: Supplementary Information — Supplementary Figures, Supplementary Tables, Supplementary Notes and Supplementary References [file ncomms14235-s1.pdf]

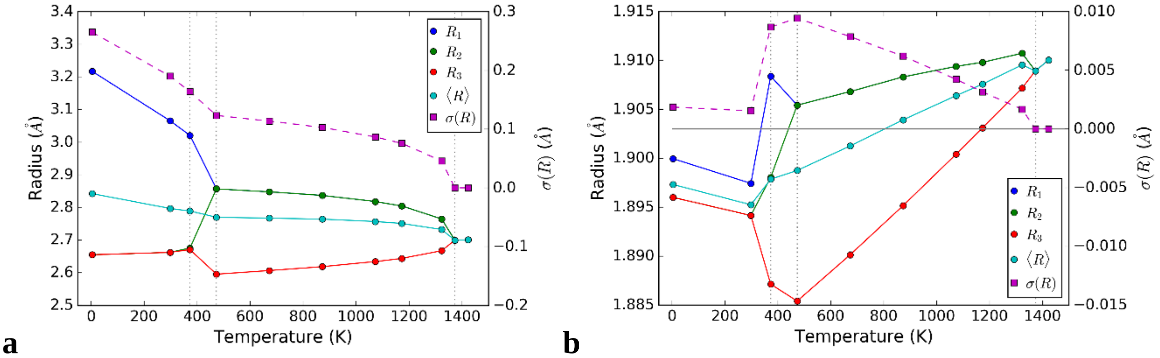

Supplementary Figure 1. Ellipsoid analysis of structural transitions in $CeAlO_3$ perovskite. (a) and **(b)** Variations of the ellipsoid radii, their average <*R*> and standard deviation σ(*R*) for the CeO_{12} and AlO_6 polyhedra respectively in $CeAlO_3$. The σ(*R*) variations at the $R\bar{3}c \rightarrow Pm\bar{3}m$ transition are similar to those of $LaAlO_3$. The I4/mcm → Imma → $R\bar{3}c$ change affects the CeO_{12} and AlO_6 polyhedra differently. $\sigma(R)_{Ce}$, equivalent to the AlO_6 tilt angle, shows an additional increase on cooling below the 473 K transition, whereas the AlO_6 octahedra become more regular with a decrease in $\sigma(R)_{Al}$.

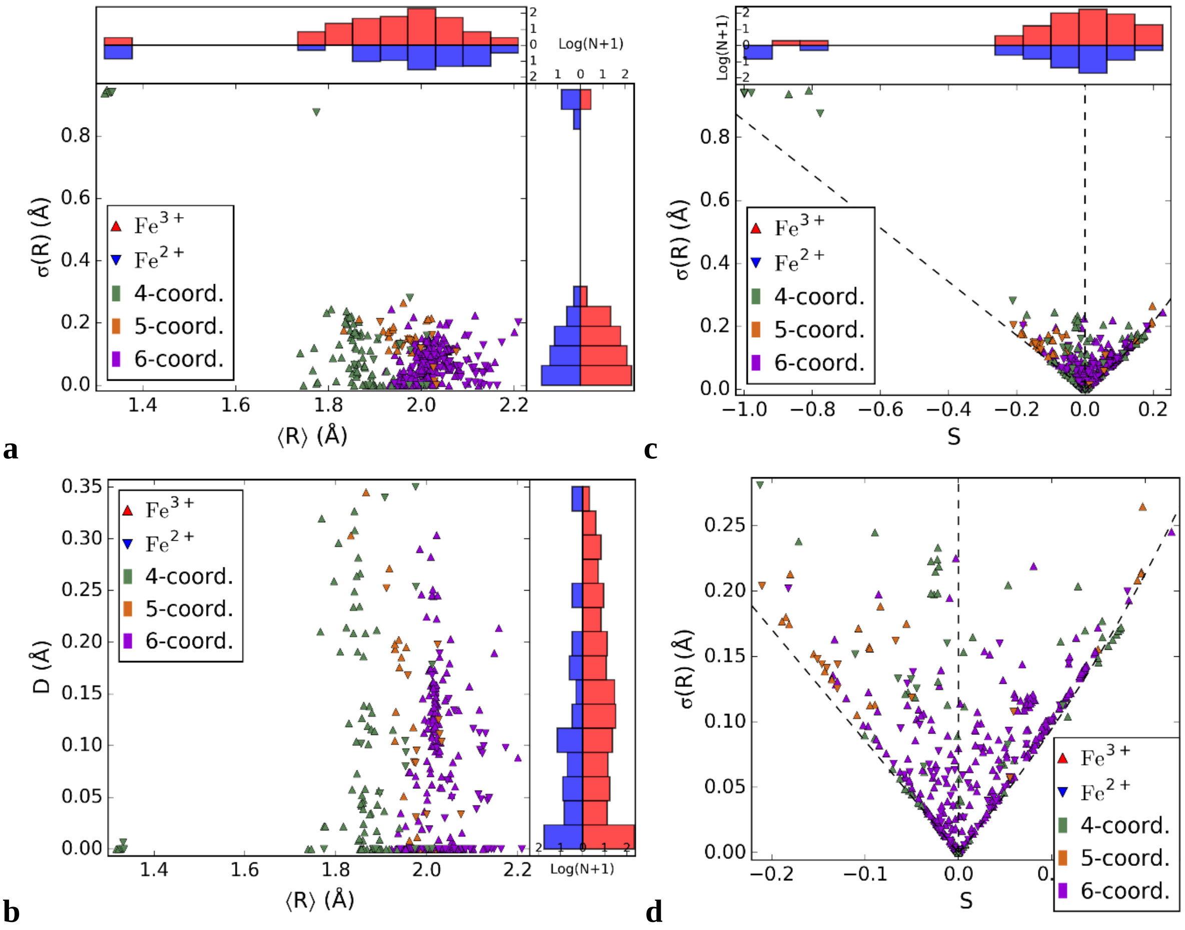

Supplementary Figure 2. Ellipsoid parameters for FeO_n polyhedra. Plots of ellipsoidal parameters for 499 FeO_n polyhedra in iron oxides (98 for Fe^{2+} and 401 for Fe^{3+}) with Fe coordination numbers between n = 4 and 6 taken from ICSD. Histograms show distributions as log(N+1) for N polyhedra within each interval. **(a)** Standard deviation σ(R) vs. mean ellipsoid radius <R>. **(b)** Displacements D of the cations from their ellipsoid centres vs. <R>. **(c)** σ(R) vs. shape parameter S showing limits for oblate ($S < 0$), geometrically scalene ($S = 0$) and prolate ($S > 0$) ellipsoids taking R_2 = 1.81 Å. **(d)** expansion for plot (c) for the region near $S = 0$.

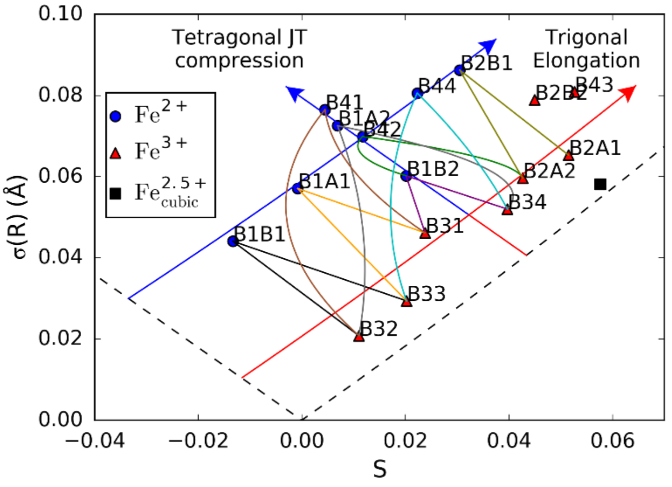

Supplementary Figure 3. Radius standard deviation σ(*R*) against shape parameter *S* for octahedra in the low temperature structure of magnetite, Fe_3O_4. Sites are labelled as in ref. 1. Each Fe^{2+} is connected to two other cations (usually Fe^{3+} ions) forming linear 3-atom trimeron units as shown by the lines. It is notable that the cations within each trimeron generally have similar values of prolate trigonal elongation. These subtle correlated distortions are brought out by the ellipsoidal approach and were not evident in previous analysis of the structure. Two Fe^{3+} ions (B2B2 and B43) do not participate in trimeron formation and are offset from the rest of the distribution at the upper right of the plot.

Supplementary Table 1: Shape parameter sign changes at perovskite phase transitions. Low temperature (LT) and high temperature (HT) space groups and signs of the shape parameter [$s(S)$] for A and B site ellipsoids at symmetry-disallowed and symmetry-allowed phase transitions between perovskite superstructures are shown.

Phase	T_c (K)	Space group		$s(S)_A$		$s(S)_B$		Ref.
		LT	HT	LT	HT	LT	HT	
Symmetry-disallowed								
$CeAlO_3$	373	I4/mcm	Imma	+	+	+	-	2
$CeAlO_3$	473	Imma	$R\bar{3}c$	+	-	-	-	2,3
$PrAlO_3$	205	Imma	$R\bar{3}c$	+	-	+	-	4
$BaCeO_3$	673	Imma	$R\bar{3}c$	+	-	-	-	5,6
$LaFeO_3$	1228	Pnma	$R\bar{3}c$	+	-	+	-	7
$PrNiO_3$	700	Pnma	$R\bar{3}c$	+	-	+	-	8
$LaCrO_3$	540	Pnma	$R\bar{3}c$	+	-	+	-	9
$LaGaO_3$	425	Pnma	$R\bar{3}c$	+	-	-	-	10
$BaPbO_3$	573	Imma	I4/mcm	+	+	+	-	11
$BaTbO_3$	280	Imma	I4/mcm	+	+	+	-	12
$CaTiO_3$	1498	Pnma	I4/mcm	+	+	+	-	13
$SrHfO_3$	870	Pnma	Cmcm	+	+ +	-	+	14
$NaNbO_3$	793	Pbcm	Cmcm	- +	+ -	+	+	15,16
$BaTiO_3$	183	R3m	Amm2	+	-	+	-	17
$BaTiO_3$	278	Amm2	P4mm	-	+	-	+	17
Symmetry-allowed								
$SrZrO_3$	1100	Cmcm	I4/mcm	+	+	-	-	18
$SrHfO_3$	1000	Cmcm	I4/mcm	+ +	+	+	-	14
$NaNbO_3$	848	Cmcm	P4/mbm	+ -	+	+	-	15
$NaTaO_3$	835	Cmcm	P4/mbm	+ +	+	-	-	19
$NaMgF_3$	1047	Pnma	P4/mbm	+	+	+	-	20
$BaCeO_3$	563	Pnma	Imma	+	+	-	-	5,6
$PrAlO_3$	150	C2/m	Imma	+	+	+	+	4

Supplementary Note 1. Symmetry-disallowed transitions in perovskites

Supplementary Table 1 shows signs of the shape parameter *S* around symmetry- disallowed and allowed transitions between perovskite superstructures.

$CeAlO_3$ and $PrAlO_3$ exhibit reported structural transitions;

I4/mcm (373 K)→ Imma (473 K)→ R$\bar{3}$c (1373 K)→ Pm$\bar{3}$m for $CeAlO_3$,[2] and

I2/m (150 K)→ Imma (205 K) → R$\bar{3}$c (~1860 K)→ Pm$\bar{3}$m for $PrAlO_3$.[4]

It is unclear whether the assigned low temperature symmetries of I4/mcm for $CeAlO_3$ and I2/m for $PrAlO_3$ are describing the same phase or not, but both materials show a symmetry-disallowed orthorhombic Imma → rhombohedral R$\bar{3}$c transition. Thermal variations of the $CeAlO_3$ ellipsoid radii, their average $\langle R \rangle$ and standard deviation $\sigma(R)$ are shown in Supplementary Fig. 1.

Transitions from orthorhombic Pbcn or Pnma symmetry to Cmcm are more complex as the latter structure has two independent A cation sites, but changes in either $s(S)_A$ or $s(S)_B$ are still observed.

Supplementary Note 2. Iron Oxides

Ellipsoidal analysis provides a useful approximation for comparing the distortions and shapes of polyhedra independent of coordination number and geometry. These aspects are illustrated with reference to iron oxide polyhedra from 390 structures in the ICSD (499 polyhedra in total) where 98 Fe^{2+} and 401 Fe^{3+} sites have coordination numbers between 4 and 6. Plots and histograms are in Supplementary Fig. 2 and summary versions of the plots are in Fig. 4.

Supplementary Figs. 2a and 2b show plots of ellipsoid standard deviation $\sigma(R)$ against mean radius $\langle R \rangle$ and shape parameter S. The $\sigma(R)$ vs. $\langle R \rangle$ distribution in Supplementary Fig. 2a shows a large cluster of points at $\langle R \rangle \approx d$ and $\sigma(R) \approx 0$, where d is an average Fe-O distance, corresponding to the large number of reported regular tetrahedra and octahedra. Ideal values based on 6-coordinate ionic radii are d = 2.18 and 2.05 Å for high spin Fe^{2+} and Fe^{3+} respectively, and the displacement between the histograms shown reflects this size difference. Fe^{3+} is often found in tetrahedral environments as shown by the collection of points near $d \approx$ 1.85 Å The $\sigma(R)$ vs. $\langle R \rangle$ plot draws attention to highly anisotropic polyhedra which have $\langle R \rangle$ values far from d and large values of $\sigma(R)$. FeO_4 square planes have $\langle R \rangle = (2/3)d \approx 1.4$ Å and $\sigma(R) = (\sqrt{2}/3)d \approx 1.0$ Å, and the cluster of points at $\sigma(R) > 0.8$ Å show this coordination, with flat square planes at $\langle R \rangle \approx 1.4$ Å and a more distorted variant at $\langle R \rangle = 1.8$ Å on Supplementary Fig. 2a.

The magnitudes D of the displacement vector for Fe relative to the ellipsoid centre of FeO_n polyhedra are plotted against $\langle R \rangle$ in Supplementary Fig. 2b. The off-centre d^5 effect is apparent but is seen more clearly for the analysis of 6-coordinate polyhedra in Fig. 5c, as described in the main paper.

The plot of $\sigma(R)$ *vs.* ellipsoid shape S in Supplementary Figs. 2c and 2d is another useful way to observe the spectrum of iron oxide polyhedra. S varies from near -1 for square planar complexes in the oblate distortion limit through many distorted and regular tetrahedra and octahedra at $S \approx -0.2$ to 0.3 (the distribution for FeO_6 polyhedra alone is in Fig. 5e). The V-shaped appearance of the distribution results from the most symmetric ellipsoid shapes imposing lower limits on σ(R); oblate coordinations have $\sigma(R) = -(\sqrt{2}/3)R_2S$ while prolate ellipsoids have $\sigma(R) = (\sqrt{2}/3)R_2S/(1-S)$. These limits are shown on the plot taking the median ellipsoid radius to be R_2 = 1.81 Å. The line for geometrically scalene ellipsoids, which have S = 0 but are non-spherical with $R_3/R_2 = R_2/R_1$ is also shown. This coordination with two long, two medium, and two short Fe-O bonds is rather rare compared to oblate and prolate coordinations.

The $\sigma(R)$ *vs.* ellipsoid shape S plot for magnetite in Fig. 4f shows remarkable structure giving insights into the electronic distortion. The same plot is reproduced as Supplementary Fig. 3 to show how distortions within trimerons are correlated.

Supplementary References

1 Senn, M. S., Wright, J. P. & Attfield, J. P. Charge order and three-site distortions in the Verwey structure of magnetite. *Nature* **481**, 173-176 (2012).

2 Fu, W. & IJdo, D. "Unusual" phase transitions in $CeAlO_3$. *J. Solid State Chem.* **179**, 2732-2738 (2006).

3 Howard, C. J., Kennedy, B. J. & Chakoumakos, B. C. Neutron powder diffraction study of rhombohedral rare-earth aluminates and the rhombohedral to cubic phase transition. *J. Phys.: Condens. Matter* **12**, 349-365 (2000).

4 Carpenter, M. A., Howard, C. J., Kennedy, B. J. & Knight, K. S. Strain mechanism for order-parameter coupling through successive phase transitions in $PrAlO_3$. *Phys. Rev. B* **72**, 024118 (2005).

5 Knight, K. Structural phase transitions in $BaCeO_3$. *Solid State Ionics* **74**, 109-117 (1994).

6 Knight, K. Structural phase transitions, oxygen vacancy ordering and protonation in doped $BaCeO_3$: results from time-of-flight neutron powder diffraction investigations. *Solid State Ionics* **145**, 275-294 (2001).

7 Selbach, S. M., Tolchard, J. R., Fossdal, A. & Grande, T. Non-linear thermal evolution of the crystal structure and phase transitions of $LaFeO_3$ investigated by high temperature X-ray diffraction. *J. Solid State Chem.* **196**, 249-254 (2012).

8 Huang, T., Parrish, W., Toraya, H., Lacorre, P. & Torrance, J. High-temperature crystal structures of orthorhombic and rhombohedral $PrNiO_3$. *Mater. Res. Bull.* **25**, 1091-1098 (1990).

9 Oikawa, K., Kamiyama, T., Hashimoto, T., Shimojyo, Y. & Morii, Y. Structural Phase Transition of Orthorhombic $LaCrO_3$ Studied by Neutron Powder Diffraction. *J. Solid State Chem.* **154**, 524-529 (2000).

10 Marti, W., Fischer, P., Altorfer, F., Scheel, H. J. & Tadin, M. Crystal structures and phase transitions of orthorhombic and rhombohedral $RGaO_3$ (R=La, Pr, Nd) investigated by neutron powder diffraction. *J. Phys.: Condens. Matter* **6**, 127-135 (1994).

11 Fu, W., Visser, D., Knight, K. & IJdo, D. High-resolution neutron powder diffraction study on the phase transitions in $BaPbO_3$. *J. Solid State Chem.* **180**, 1559-1565 (2007).

12 Fu, W., Visser, D., Knight, K. & IJdo, D. Temperature-induced phase transitions in $BaTbO_3$. *J. Solid State Chem.* **177**, 1667-1671 (2004).

13 Ali, R. & Yashima, M. Space group and crystal structure of the Perovskite $CaTiO_3$ from 296 to 1720 K. *J. Solid State Chem.* **178**, 2867-2872 (2005).

14 Kennedy, B. J., Howard, C. J. & Chakoumakos, B. C. High-temperature phase transitions in $SrHfO_3$. *Phys. Rev. B* **60**, 2972-2975 (1999).

15 Darlington, C. N. W. & Knight, K. S. High-temperature phases of $NaNbO_3$ and $NaTaO_3$. *Acta Crystallogr.* **B55**, 24-30 (1999).

16 Johnston, K. E., Tang, C. C., Parker, J. E., Knight, K. S., Lightfoot, P. & Ashbrook, S. E. The Polar Phase of $NaNbO_3$: A Combined Study by Powder Diffraction, Solid-State NMR, and First-Principles Calculations. *J. Am. Chem. Soc.* **132**, 8732-8746 (2010).

17 Kwei, G. H., Lawson, A. C. & Billinge, S. J. L. Structures of the Ferroelectric Phases of Barium Titanate. *J. Phys. Chem.* **97**, 2368-2377 (1993).

18 Kennedy, B. J., Howard, C. J. & Chakoumakos, B. C. High-temperature phase transitions in $SrZrO_3$. *Phys. Rev. B* **59**, 4023-4027 (1999).
19 Kennedy, B. J., Prodjosantoso, A. K. & Howard, C. J. Powder neutron diffraction study of the high temperature phase transitions in $NaTaO_3$. *J. Phys.: Condens. Matter* **11**, 6319-6327 (1999).
20 Knight, K. S., Price, G. D., Stuart, J. A. & Wood, I. G. High-temperature structural phase transitions in neighborite: a high-resolution neutron powder diffraction investigation. *Phys. Chem. Minerals* **42**, 45-52 (2014).
